# 346. Head to Toe Imaging of Complications and Sequelae of COVID- 19 and COVID-19 Vaccination

**DOI:** 10.1093/ofid/ofab466.547

**Published:** 2021-12-04

**Authors:** Deepti H Vijayakumar, Deepali Saxena, Rajesh V Helavar, Raghavendra Tirupathi

**Affiliations:** 1 Columbia Asia Referral Hospital, Bangalore, Bengaluru, Karnataka, India; 2 Columbia Asia Referral Hospital, Bengaluru, Karnataka, India; 3 WellSpan Health, Chambersburg, Pennsylvania

## Abstract

**Background:**

COVID 19 is associated with a hypercoagulable state with cytokine storm syndrome and thrombocytopenia leading to complications across various systems. COVID-19 infection, its treatment, resultant immunosuppression, and pre-existing comorbidities have made patients vulnerable to secondary infections

**Methods:**

We systematically reviewed COVID-19 cases between Jan to May 2021 for pulmonary and extrapulmonary complications. Patients with recent COVID-19 vaccination and neurological symptoms were also included.

Figure 1. “Black turbinate” sign of mucormycosis

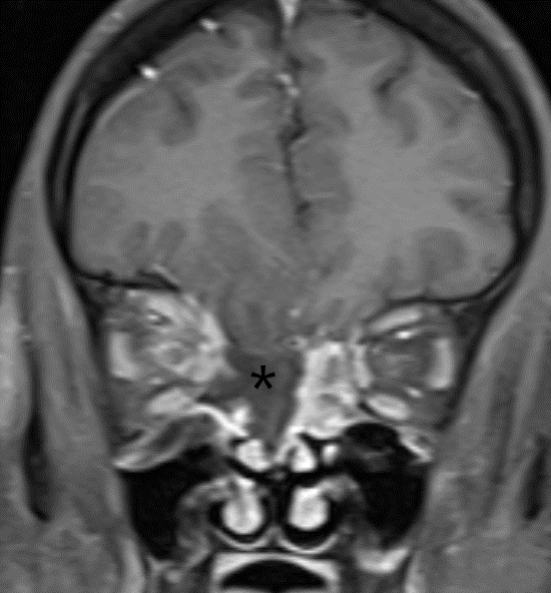

Contrast enhanced coronal T1 FS images of paranasal sinuses shows necrotic non-enhancing right superior and middle turbinates (*) with extension into the right orbital fat.

FIGURE 2 - A composite image of Coronal CT of upper abdomen in arterial phase and lung bases in lung window showing wedge showing right renal infarcts (line arrow) due to inferior polar artery thrombosis and ground glass opacities (solid arrow) in lung bases.

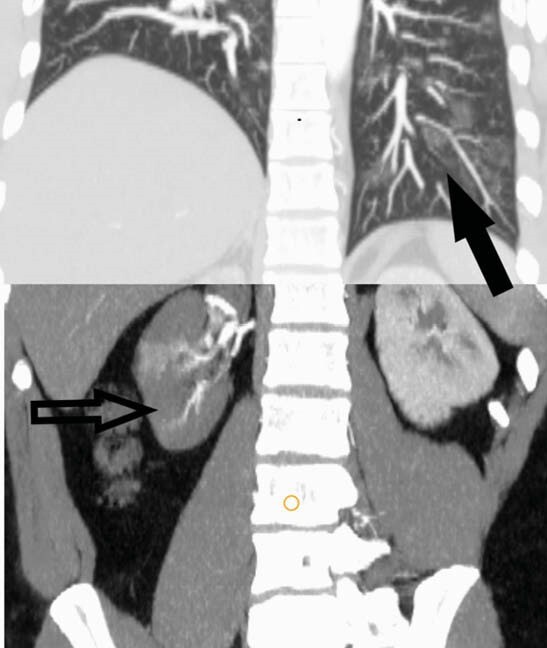

**Results:**

*Neurological complications*: Neurological complications include ischemic and haemorrhagic strokes. Other complications are encephalopathy, encephalitis, Guillain-Barré syndrome, acute hemorrhagic necrotizing encephalopathy. Demyelination and radiculopathies are seen as post vaccination complications. *Mucormycosis*: Unprecedented high rate of invasive fungal sinusitis in association with COVID -19 is reported from the Indian subcontinent. This has a propensity for intra orbital and intracranial extension. *COVID -19 associated coagulopathy*: COVID -19 is a pro-inflammatory hypercoagulable state. Pulmonary thromboembolism, deep venous thrombosis and catheter related thrombosis are well documented. *Cardiac complications*: Cardiac manifestations include Myocardial Injury with non-obstructed coronary arteries (MINOCA), myocarditis, myocardial ischemia, cardiomyopathy. *Pulmonary complications and sequelae of COVID -19*: Progression of lung injury to ARDS during the initial phase and fibrosis of parenchyma in the recovery phase. Spontaneous pneumomediastinum, pneumatoceles and pneumothorax and secondary infections are identified in our study. *COVID- 19 associated gastrointestinal complications:* Patients evaluated for renal colic, pancreatitis, cholecystitis showed, ground glass opacities or subpleural bands in typical Covid-19 distribution. COVID-19 may lead of acute kidney and bowel injury due to arterial thrombosis. *COVID - 19 associated myonecrosis*: Ischemia of the small caliber vessels may result in myonecrosis.

FIGURE 3 - Coronal STIR image shows thickened and hyperintense trunks and divisions of the right brachial plexus suggestive of plexopathy in a COVID -19 patient with H/O recent COVID-19 vaccination.

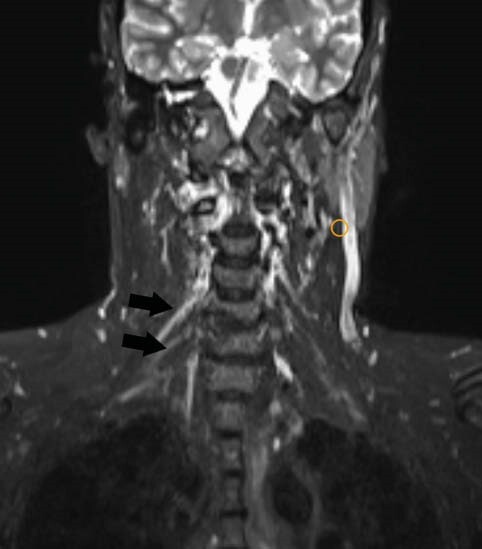

Figure 4. Axial CT chest section in lung window showing pneumothorax (white arrow) and pneumatocele ( grey arrow) with peripheral ground glass opacities and consolidations in both lungs.

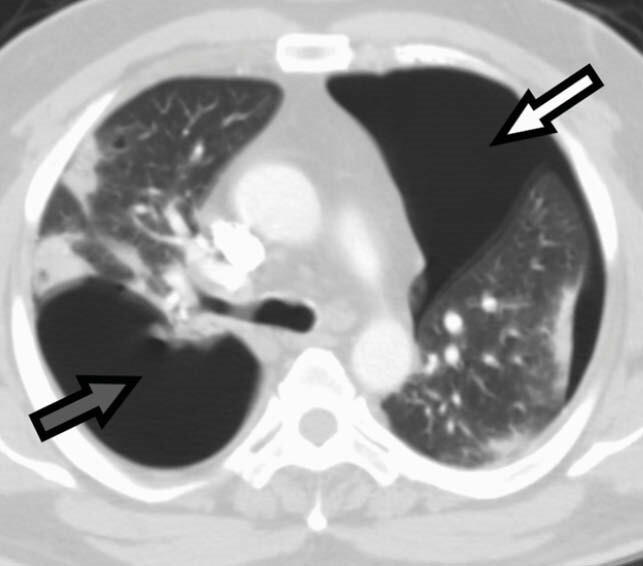

**Conclusion:**

Awareness of these unusual manifestations will facilitate an early diagnosis, improve management and help reduce morbidity and mortality

**Disclosures:**

**All Authors**: No reported disclosures

